# ANGPTL-4 is Associated with Obesity and Lipid Profile in Children and Adolescents

**DOI:** 10.3390/nu11061340

**Published:** 2019-06-14

**Authors:** Silvia Barja-Fernández, Cintia Folgueira, Cecilia Castelao, Verónica Pena-León, Patricia González-Saenz, Rocío Vázquez-Cobela, Concepción M. Aguilera, Mercedes Gil-Campos, Gloria Bueno, Ángel Gil, Luis A. Moreno, Manuel Ruiz-Piñon, María García-Palacios, Felipe F. Casanueva, Carlos Diéguez, Rubén Nogueiras, Rosaura Leis, Luisa M. Seoane

**Affiliations:** 1 Endocrine Physiopathology Group, Instituto Investigación Sanitaria Santiago de Compostela (IDIS), Complejo Hospitalario Universitario Santiago de Compostela (CHUS/SERGAS), 15706 Santiago de Compostela, Spain; sylvia_barfer@hotmail.com (S.B.-F.); cintiafolgueira@gmail.com (C.F.); ceciliacastelao@hotmail.com (C.C.); veritoloren@hotmail.com (V.P.-L.); pepsieg@gmail.com (P.G.-S.); manuelc.ruiz@usc.es (M.R.-P.); maria.garcia.palacios@gmail.com (M.G.-P.); 2 Pediatrics Department, GI Nutrición Pediátrica (IDIS, CHUS), Galician Human Development, Growth and Nutrition Research Unit, Universidad Santiago de Compostela (USC), 15706 Santiago de Compostela, Spain; cobela.rocio@gmail.com; 3 CIBER Fisiopatología Obesidad y Nutrición (CIBEROBN), Instituto Salud Carlos III, 28029 Madrid, Spain; caguiler@ugr.es (C.M.A.); mercedes_gil_campos@yahoo.es (M.G.-C.); gbuenoloz@yahoo.es (G.B.); agil@ugr.es (Á.G.); lmoreno@unizar.es (L.A.M.); felipe.casanueva@usc.es (F.F.C.); carlos.dieguez@usc.es (C.D.); 4 Department Physiology, Center for Research in Molecular Medicine and Chronic Diseases (CIMUS), Health Research Institute of Santiago USC-IDIS, University Santiago de Compostela, 15705 Santiago de Compostela, Spain; 5 Biochemistry and Molecular Biology II Department, University Granada, 18001 Granada, Spain; 6 Paediatric Research and Metabolism Unit, University Hospital Reina Sofia, 14004 Córdoba, Spain; 7 Pediatric Department, University Hospital Lozano Blesa, 50009 Zaragoza, Spain; 8 GENUD (Growth, Exercise, Nutrition and Development research group, Universidad de Zaragoza, Instituto Agroalimentario de Aragón (IA2) and Instituto de Investigación Sanitaria de Aragón (IIS Aragón), 50009 Zaragoza, Spain; 9 Operative Dentistry and Endodontics, USC, 15782 Santiago de Compostela, Spain; 10 Department Pediatric Surgery, University Santiago de Compostela USC, 15705 Santiago de Compostela, Spain; 11 Laboratory of Molecular and Cellular Endocrinology, University Santiago de Compostela USC, 15706 Santiago de Compostela, Spain

**Keywords:** ANGPTL-4, BMI loss, childhood, lipid profile, obesity

## Abstract

Angiopoietin-like protein 4 (ANGPTL-4) regulates lipidic metabolism and affects energy homeostasis. However, its function in children with obesity remains unknown. We investigated plasma ANGPTL-4 levels in children and its relationship with body mass index (BMI) and different lipidic parameters such as free fatty acids (FFA). Plasma ANGPTL-4 levels were analyzed in two different cohorts. In the first cohort (n = 150, age 3–17 years), which included children with normal weight or obesity, we performed a cross-sectional study. In the second cohort, which included only children with obesity (n = 20, age 5–18 years) followed up for two years after an intervention for weight loss, in which we performed a longitudinal study measuring ANGPTL-4 before and after BMI-loss. In the cross-sectional study, circulating ANGPTL-4 levels were lower in children with obesity than in those with normal weight. Moreover, ANGPTL-4 presented a negative correlation with BMI, waist circumference, weight, insulin, homeostasis model assessment of insulin resistance index (HOMA index), triglycerides, and leptin, and a positive correlation with FFA and vitamin-D. In the longitudinal study, the percent change in plasma ANGPTL-4 was correlated with the percent change in FFA, total-cholesterol and high-density lipoprotein cholesterol. This study reveals a significant association of ANGPTL-4 with pediatric obesity and plasma lipid profile.

## 1. Introduction

Angiopoietin-like protein 4 (ANGPTL-4) is mainly produced in adipose tissue and liver [1,2], and, additionally, by a broad range of tissues [3,4,5]. Of the functions performed by ANGPTL-4, the control of lipid metabolism has lately aroused great interest. ANGPTL-4 has emerged as an inhibitor of lipoprotein lipase (LPL), an enzyme that promotes the hydrolysis of circulating triglycerides (TG) associated with lipoproteins, giving rise to free fatty acids (FFA) that are captured by the surrounding tissues [6,7].

ANGPTL-4 gene codes for a native full-length glycoprotein (~45–65 kDa), which is secreted and processed by proprotein convertases releasing the N-terminal and the monomeric C-terminal fragments [7,8]. The ANGPTL-4 cleavage may be dependent on the tissue due to tissue-specific expression of proprotein convertases [8]. The complete form is mostly released from the adipose tissue while the N-terminal portion is primarily produced in the liver [9]. Both ANGPTL-4 truncated forms and full-length protein are present in the bloodstream [9,10]. Several studies indicate that only the full-length and N-terminal portion inhibit LPL, whereas the C-terminal fragment participates in others functions, such as angiogenesis and protection of cancer cells against apoptosis and anoikis [11,12]. However, a recent study reveals that the C-terminal domain has additional lipolytic and thermogenic properties [13]. Since ANGPTL-4 can be secreted by a wide variety of tissues and in different forms, the biological functions of this protein might be more complex than expected.

Recently, in preclinical models, an increasing number of studies have highlighted the role of ANGPTL-4 in energy homeostasis [3,5,14]. It has been described that ANGPTL-4 production is regulated by nutritional status such as fasting [15], caloric restriction [4,9] and physical activity [4,16] in humans. However, the studies performed in humans have not been able to elucidate the role of ANGPTL-4 in obesity and controversial data have been reported. For example, a study in 2010 showed a positive correlation between ANGPTL-4 and FFA circulating levels, waist circumference, and age in patients 30–60 years of age. However, when the analysis was adjusted by age, a negative correlation was detected between ANGPTL-4 and body mass index (BMI) in the younger subjects (30–45 years) but not in the older subjects (45–60 years) [10]. In addition, other published studies show nil, positive, or negative correlations between ANGPTL-4 and BMI [14,17,18]. Supporting the role of ANGPTL-4 in obesity, associations between ANGPTL-4 gene polymorphism and a high percentage of body fat, low levels of TG, or disrupted serum lipid concentrations have been reported [19,20,21]. Furthermore, recent studies have shown a link between abnormal glucose tolerance and high ANGPTL-4 levels [22]. In support of this observation, we recently published a report on the significant involvement of ANGPTL-4 in human obesity and type 2 diabetes and its participation in long-term body weight modifications [23]. In addition, the effect of ANGPTL-4 in increasing lipolysis has been described [3,24]. Lipolysis induces the hydrolysis of triacylglycerols, releasing FFA into the circulation [25]. In this context, the relation between ANGPTL-4 and FFA levels might indicate the possible effect of ANGPTL-4 on obesity.

Recent studies have highlighted the strong association between ANGPTL-4 levels and obesity in adults. However, in children, the precise role of ANGPTL-4 in obesity remains unknown.

Thus, the main objective of the present study was to ascertain whether ANGPTL-4 levels are altered in children with obesity and determine a possible role of this protein in the BMI reduction achieved after obesity-oriented procedures in pediatric patients, suitable to be used as a potential biomarker for the risk of obesity in children. Additionally, the relation between ANGPTL-4 and circulating levels of the main biochemical parameters known to be affected in the obese state (FFA, cholesterol, TG, insulin, vitamin D, and leptin) were determined in this population.

## 2. Materials and Methods

### 2.1. Cross-Sectional Study

To determine if the state of obesity is related to variations in ANGPTL-4 levels in children, the first cohort of patients were recruited including a total of 150 Caucasian children and adolescents (3–17 years). This cohort was classified according to BMI as obese (n = 77) or normal weight (n = 73) utilizing the international BMI cut-off points defined by Cole et al. [26] to compare the levels of ANGPTL-4 in children with obesity with respect to those having normal weight. Enrolment into the study occurred between 2006 and 2016. The patients were enlisted from Investigation in Nutrition, Growth and Human Development of Galicia Unit (Clinic University Hospital of Santiago de Compostela), Molecular Biology and Biochemistry II Department (University of Granada), Pediatric Endocrinology Unit (University Hospital Reina Sofia of Córdoba and University Hospital Lozano Blesa of Zaragoza) and Public Health Department (University of Zaragoza).

### 2.2. Longitudinal Study

To determine if the changes in BMI achieved after an intervention to reduce weight affected ANGPTL-4 levels, the second cohort of children, independent from the one used for the cross-sectional study, was recruited in the longitudinal study (5–18 years). This cohort comprised children with obesity (n = 20) who were followed for 2 years after participating in an intervention to reduce weight. The intervention consisted of providing general counselling about a healthy lifestyle to each participant at the beginning of the study. This included nutritional (healthy diet) and physical activity guidelines that individuals must follow during the follow-up period to achieve weight loss and the corresponding BMI change. Specifically, it gave patients recommendations for healthy guidelines based on the food pyramid of the Mediterranean diet with special mention of the intake of more than three portions of fish a week, five portions of fruit and vegetables a day and use of olive oil. In addition, the promotion of more than one hour a day of moderate physical activity and less than two hour a day of screen use. After 2 years of follow up, BMI loss was achieved.

### 2.3. Ethical Considerations

Subjects were excluded if they had received any medication or suffered any chronic disorder that might interfere with the study results. Both studies, cross-sectional and longitudinal, were conducted in accordance with the Declaration of Helsinki and all procedures were authorized by the Central Ethics and Research Committee of Galicia Autonomous Community (code 2013/256). The acceptance of all children was obtained and the written informed consent was signed by the parents.

### 2.4. Clinical Examination and Blood Sampling

The anthropometric parameters were collected in the morning by a single pediatrician with the subject in underwear and without shoes. Body weight was recorded with a digital electronic balance to the nearest 0.1 kg (Digital electronic balance Seca mod. 813. gmbh & CO) and height with a calibrated wall-mounted stadiometer to the nearest 0.1 cm (stadiometer Seca mod. 213. gmbh & CO). To calculate the BMI, the weight (kg) was divided by height squared (m^2^).

Clinical examination was performed by coached pediatricians employing settled out methods. The pubertal period was evaluated by a gender-specific questionnaire in accordance with Tanner’s criteria.

Blood samples were collected between 08:00 and 09:00 after 12 h of overnight fasting. The blood samples were gathered into tubes with EDTA (1 mg/mL). Plasma was split by centrifugation at 4 °C and stocked at −80 °C.

### 2.5. Biochemical Assays

The determination of ANGPTL-4 levels in plasma samples was carried out by a commercial enzyme-linked immunoabsorbent assays (ELISA) kit (Human ANGPTL4 ELISA kit SK00309-01; Aviscera Bioscience, Inc., Santa Clara, CA, USA), as per the Manufacturer’s instructions. This ELISA kit has been pre-validated in the bibliography and it recognizes the full-length of ANGPTL-4 [23]. Intra-assay and inter-assay variation coefficients were 4–6% and 8–10%, respectively. The assay sensitivity limit was 0.15 ng/mL.

For each sample, the absorbance was assayed in duplicate by the use of a spectrophotometric microplate reader at a wavelength of 450 nm (Epoch 2 microplate reader/Biotek Instruments, Inc., Winooski, VT, USA). The units for the results presented are ng/mL.

Plasma glucose, total cholesterol, and TG were measured in an Advia 2400 Chemistry System (Siemens Healthcare Diagnostics, Erlangen, Germany). Low-density lipoprotein (LDL) cholesterol and high-density lipoprotein (HDL) cholesterol levels were quantified with a SAS-3 Cholesterol Profile kit (Helena Biosciences Europe, Gateshead, UK). Plasma leptin was determined with a commercial ELISA kit following the instructions (DRG International, Springfield Township, NJ, USA). Plasma levels of insulin, thyroid-stimulating hormone (TSH), free triiodothyronine (fT3), free thyroxine (fT4), estradiol, testosterone, follicle stimulating hormone (FSH) and vitamin-D were determined using a chemiluminescence immunoassay (Advia Centaur XP analyzer; Siemens Healthcare Diagnostics, Erlangen, Germany). Plasma insulin-like growth factor 1 (IGF-1) was analyzed with a chemiluminescence immunoassay (Immulite 2000 analyzer; Siemens Healthcare Diagnostics, Erlangen, Germany). Circulating FFA was determined using a colorimetric kit following the manufacturer’s instructions (Wako Chemicals GmbH, Neuss, Germany).

### 2.6. ANGPTL-4 Pre-Adsorption

The pre-adsorption of ANGPTL-4 was performed as described by Barja-Fernández et al. [23]. ANGPTL-4 concentration of the pre-adsorbed sample was measured by ELISA to determine the specificity of the ELISA kit used.

### 2.7. Statistical Analysis

SPSS version 20.0 software (SPSS, Chicago, IL, USA) was employed for the statistical analyses. Data are presented as mean (standard deviation, SD). Variations in gender and sexual development distribution were determined by chi-square test. If necessary, the log transformation of the data was performed to get a satisfactory fit to the normal distribution or variance homogeneity. To study the effect of the obese state and the follow-up intervention, the statistical tests utilized were the Student’s unpaired and paired t-test or Wilcoxon test when appropriate. If the data followed a normal distribution, the relation among variables was assessed by the use of Pearson’s correlation coefficient, whereas, when the data followed a non-normal distribution, the coefficient employed was the Spearman´s rank test.

The relationship between ANGPTL-4 and the anthropometric, biochemical and hormonal variables have been assessed by the multivariate linear regression. The criterion for choosing the best model relied on the Akaike Information Criterion (AIC), assuming that models with multicollinearity were refused. The best model was achieved using an automatic staggered sorting method based on bidirectional elimination, a combination of forward and backward sorting, and verification of the presence of variables for exclusion or inclusion at each step. In the final stage of the procedure, multicollinearity in the explanatory variables was ascertained by applying the Variance Inflation Factor (VIF), and no problems were observed: the VIF for all covariates was lower than 3 (problems appear when the VIF is greater than 5). Levels of statistical significance were set at *p* < 0.05.

## 3. Results

### 3.1. Plasma ANGPTL-4 Levels Are Reduced in Children and Adolescents with Obesity and Associated with Obesity-Related Parameters

The anthropometric and biochemical characteristics of the cross-sectional study population are summarized in Table 1. Individuals with normal weight and obesity did not differ according to age, gender, or sexual development. Individuals with obesity exhibited higher body weight, height, BMI, waist circumference, homeostasis model assessment of insulin resistance index (HOMA index), plasma insulin, leptin, TG, and fT4 while glucose, HDL-cholesterol and vitamin-D levels were reduced compared with age-matched and gender-matched individuals with normal weight (Table 1). When the population was classified by gender, boys with obesity had higher concentrations of TSH and fT4 than boys with normal weight, whereas girls with obesity presented lower levels of glucose and vitamin-D than those girls with normal weight (Table S1). When the population was classified by sexual development, both children (pre-pubertal stage) and adolescents (pubertal stage) with obesity showed higher body weight, height, BMI, waist circumference, HOMA index, and plasma insulin, and leptin than those with normal weight. However, only pre-pubertal children with obesity exhibited a diminution of glucose concentration with respect to those with a normal weight that was not found in adolescent subjects. In addition, a decrease in vitamin-D levels and an increase in FSH were found only in adolescents with obesity compared to those with normal weight (Table S2).

**Figure 1:**
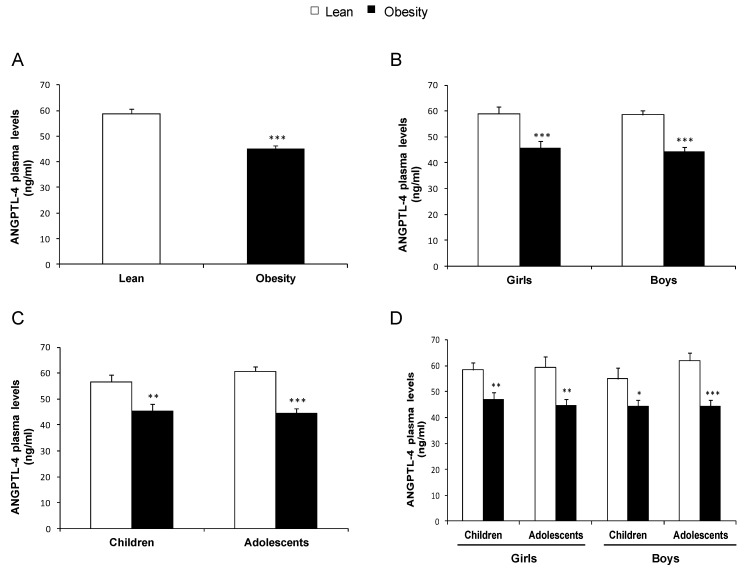
ANGPTL-4 circulating levels of the cross-sectional study population: (**A**) ANGPTL-4 concentration in individuals with normal weight and obesity; (**B**) ANGPTL-4 levels according to gender; (**C**) ANGPTL-4 concentration according to the pubertal stage; and (**D**) ANGPTL-4 levels according to gender and pubertal stage. Data are expressed as mean ± SEM. * *p* < 0.05, ** *p* < 0.01, *** *p* < 0.001.

In the cross-sectional study population, the mean value of ANGPTL-4 concentration was 51.8 (1.2) ng/mL, with values ranging from 18.3 ng/mL to 101.0 ng/mL. Circulating levels of ANGPTL-4 were lower in individuals with obesity than in those with normal weight in the total population (Figure 1A). To explore the effect of gender-dependent differences, the data were independently analyzed in girls and boys. In both girls and boys with obesity, ANGPTL-4 levels were significantly decreased (Figure 1B). To determine whether sexual development affected in the regulation of ANGPTL-4 concentration, we analyzed the data according to the puberty stage. Both children (pre-pubertal stage) and adolescents (pubertal stage) with obesity showed reduced circulating ANGPTL-4 levels with respect to those with normal weight (Figure 1C). Finally, we analyzed the data as a function of pubertal stage separately in girls and boys and found that the decrease in circulating ANGPTL-4 in individuals with obesity was independent of the gender and the pubertal stage (Figure 1D).

In addition, we performed a specificity study of the ELISA kit to validate the kit used by measuring the preadsorption samples. We found that ANGPTL-4 concentration of preadsorbed samples was reduced by 99% in comparison with samples without preadsorption (Table S3).

**Figure 2:**
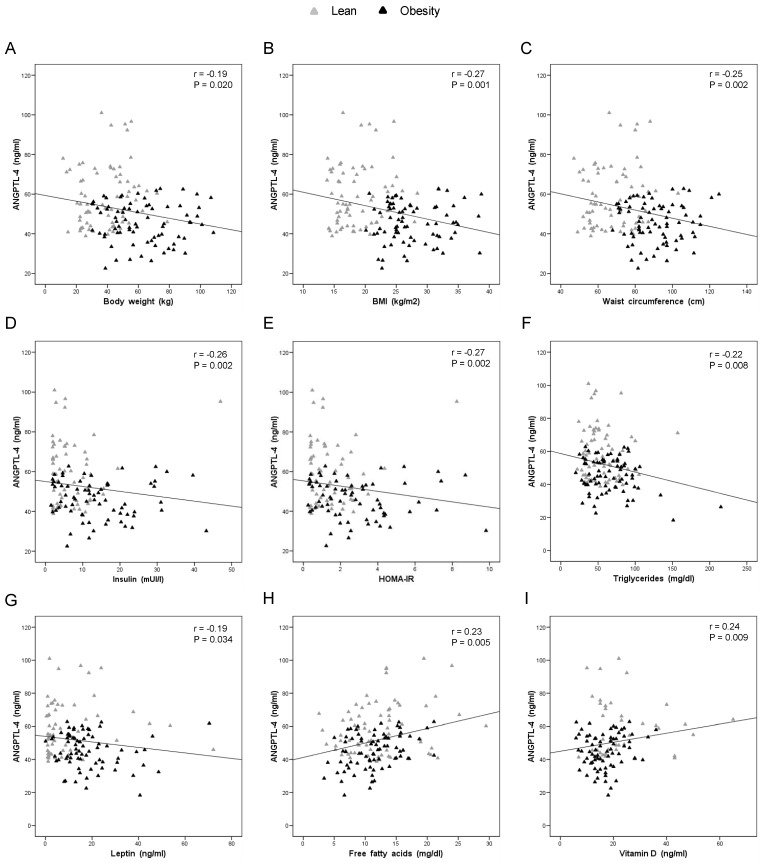
Correlation analysis of cross-sectional study population. Bivariate correlation between plasma ANGPTL-4 levels and: body weight (**A**); BMI (**B**); waist circumference (**C**); insulin (**D**); HOMA index (**E**); TG (**F**); leptin (**G**); FFA (**H**); and vitamin-D (**I**).

Taking into account that gender-dependent differences were not found in the present work, the correlation of plasma ANGPTL-4 levels with anthropometric and biochemical parameters was assessed in the total population. The correlation study revealed that ANGPTL-4 was negatively correlated with body weight, BMI, waist circumference, insulin, HOMA index, TG and leptin (Figure 2A–G). Similarly, ANGPTL-4 was positively correlated with FFA and vitamin-D levels (Figure 2H,I and Table S4). When the correlation study was performed independent of the obesity diagnosis, it was found that all correlations between ANGPTL-4 and anthropometric and biochemical characteristics were absent in individuals with normal weight. However, in individuals with obesity, the negative and positive correlation with TG and FFA, respectively, were still persistent. In addition, a negative correlation between ANGPTL-4 and total cholesterol was observed in individuals with obesity (Table S5).

In the multiple linear regression analysis, the optimal model that best explained the ANGPTL-4 data included obesity diagnostic, FFA, LDL-cholesterol, vitamin-D and total cholesterol, with an adjusted R^2^ value of 0.30 (Table 2).

### 3.2. Impact of BMI Loss and Plasma Lipid Changes on ANGPTL-4 Circulating Levels

**Figure 3:**
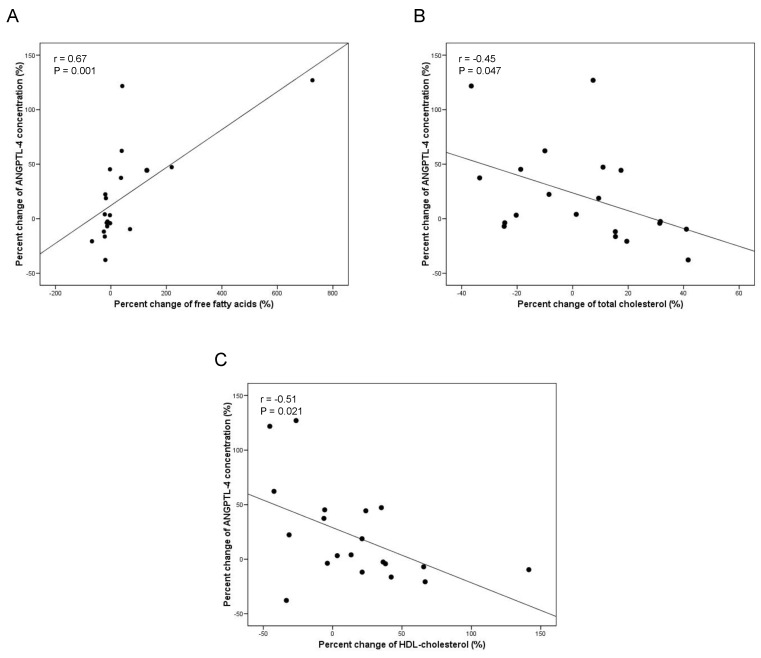
Correlation analysis of the longitudinal study population. Bivariate correlation between ANGPTL-4 percent change and percent change of: FFA (**A**); total cholesterol (**B**); and HDL-cholesterol (**C**).

The principal anthropometric and biochemical parameters recorded in the longitudinal study participants are shown in Table 3. The two years of lifestyle intervention significantly reduced BMI, fat mass, percent of fat mass, and waist circumference of the patients with obesity. BMI loss caused by the healthy lifestyle intervention did not produce significant alterations in ANGPTL-4 circulating levels (Table 3). Nevertheless, correlation studies showed that the percent change of plasma ANGPTL-4 was positively correlated with the percent change of FFA and negatively with the percent change of total cholesterol and HDL-cholesterol (Figure 3 and Table S6). These results were additionally supported by the results of the multiple linear regression study. The optimal model that best explained the percent change of circulating ANGPTL-4 included the percent change of FFA, HDL-cholesterol, total cholesterol and TG, with a corrected R^2^ value of 0.67 (Table S7).

## 4. Discussion

To the best of our knowledge, the present study demonstrates for the first time that ANGPTL-4 circulating levels are affected in patients with obesity and also by long-term changes in plasma lipid profile in the pediatric age group. The main findings are as follows: First, circulating levels of ANGPTL-4 were decreased in subjects with obesity compared with individuals with normal weight, independent of the gender and pubertal stage. Second, circulating levels of ANGPTL-4 were negatively correlated with the main markers of adiposity such as BMI, waist circumference, and weight; metabolic parameters such as insulin, HOMA index, TG, and leptin; and positively correlated with FFA and vitamin-D levels. Third, in subjects with obesity and BMI loss, the percent change of ANGPTL-4 was positively correlated with the percent change of FFA and negatively with the percent change of total cholesterol and HDL-cholesterol. The most interesting finding was that changes in plasma ANGPTL-4 levels were more affected by changes in plasma lipid levels than by changes in BMI.

ANGPTL-4 has been recently proposed as a key factor regulating lipid metabolism by its inhibitory role on LPL activity [7]. Moreover, ANGPTL-4 is a component of the machinery modulating HDL metabolism and function, besides regulating lipoprotein metabolism [7,21]. Pre-clinical studies showed that administration of ANGPTL-4 induced a diminution in food intake and body weight gain while raising energy expenditure [5]. Several metabolic actions of ANGPTL-4 were described such as the effect on adipose tissue lipolysis and glucose homeostasis regulation [7]. Lately, it has been demonstrated that ANGPTL-4 produced by brown adipose tissue plays a relevant role in the control of lipid and glucose metabolism and thermogenesis regulation [27]. Although various studies have tried to find if ANGPTL-4 would be involved in obesity and the associated comorbidities, there is a lack of consensus in this issue.

Recent studies have shown that plasma levels of ANGPTL-4 is higher in individuals with obesity and type 2 diabetes [22,28,29], and it is positively associated with obesity-related parameters in adult populations [18,28]. Accordingly, a very recent paper from our group shows that plasma ANGPTL-4 concentration is significantly increased in patients with obesity, especially in those with altered glucose tolerance [23]. In contrast, other studies have not found a relation between ANGPTL-4 and obesity [14]. Moreover, another interesting study describes that ANGPTL-4 circulating levels are decreased in the youngest individuals with obesity (35–45 years), with no differences in the eldest population [10]. In addition, a study performed on young adult twins shows that ANGPTL-4 levels decrease in those with higher body weight than in their co-twins with normal weight [17]. Overall, these discordant findings might indicate a different regulation of ANGPTL-4 with age, at least in adults.

Human obesity is characterized by an excess of adiposity which is associated with alterations in the plasma lipid profile as a consequence of the increase in adipose mass. In patients with obesity, several biochemical parameters are altered including FFA, TG, cholesterol, vitamin D, insulin, and leptin [23,30]. All recent data in adults indicate that ANGPTL-4 might have a strong role in metabolism control and might be altered in obesity. In early ages, especially in puberty, characteristic metabolic changes occur. However, in pediatric obesity, no data exist about the relationship between plasma levels of ANGPTL-4 and the main parameters related to obesity such as FFA, vitamin D, insulin, cholesterol, etc. Furthermore, no data exist about how ANGPTL-4 levels are modified after changes in BMI in childhood.

The present data show a decrease of ANGPTL-4 levels in children and adolescents with obesity and, for the first time in pediatric subjects, demonstrates a negative correlation between ANGPTL-4 and the main markers of obesity (body weight, BMI, waist circumference, HOMA index, insulin, leptin, and TG). These findings coincide with the data published by Robciuc et al. for an adult population [10,17]. However, different from adults, we show that the relation between ANGPTL-4 and obesity is independent of the age in the pediatric population. Future studies should focus on finding if these discrepancies between pediatric and adult populations are the result of differential regulation of ANGPTL-4 level in the pediatric stage as compared to in adults, as it has already been reported for other hormones like leptin [31].

Only one study focused on the pediatric age group has shown that circulating ANGPTL-4 levels are significantly lower in children and adolescents with obesity and positively correlated with peroxisome proliferator-activated receptor expression in peripheral blood mononuclear cells [32]. Accordingly, the present work shows a decrease in ANGPTL-4 levels in children and adolescents with obesity independent of the gender and pubertal stage. Moreover, a significant association between ANGPTL-4 genetic polymorphism and body fat percent in adolescents has been previously reported [19].

To the best of our knowledge, the present study for the first time reports a strong negative correlation between ANGPTL-4 levels in children and the main anthropometric (body weight, BMI, and waist circumference) and metabolic (HOMA index, insulin, leptin, and TG) parameters of obesity, thus supporting the decreased levels of ANGPTL-4 found in children with obesity.

Different studies have suggested that ANGPTL-4 stimulates lipolysis in adipocytes increasing FFA circulating levels [3,24]. Additionally, we found a positive correlation between ANGPTL-4 and FFA, which might indicate that those individuals with lower ANGPTL-4 levels present a lower lipolytic activity resulting in a higher adiposity characteristic of the obese state. Accordingly, plasma ANGPTL-4 has a positive correlation with the levels of the hormone-sensitive lipase, the main marker of lipid oxidation, and with plasma FFA in young adults, supporting a role for ANGPTL-4 in inducing lipolysis in humans [14,17].

It is widely known that plasma vitamin-D is determined by environmental factors, especially by sunlight exposure [33]. We recently published that vitamin-D status in pediatric age is associated with adiposity in addition to gender, puberty, and age [34]. The present study is the first in reporting that plasma ANGPTL-4 and vitamin-D are positively associated. Therefore, future studies should be designed to understand the relation between vitamin-D and ANGPTL-4.

Given that ANGPTL-4 is influenced by obesity in pediatric age, we hypothesized that its levels in plasma could be influenced by changes in BMI. In the longitudinal study, we failed to detect changes in plasma ANGPTL-4 after BMI loss. However, it is interesting to highlight that the percent change of plasma ANGPTL-4 was positively correlated with the percent change of FFA and negatively correlated with the percent change of total and HDL-cholesterol after BMI loss. Thus, these results suggest a strong association between ANGPTL-4 and circulating lipid changes in the pediatric age.

The results of the present work suggest that ANGPTL-4 might be considered as a potential biomarker for the risk of obesity in children as well as alterations on lipid and glucose metabolism as obesity co-morbidities. This may allow predicting improvements on lipid profile after interventions focused on weight loss.

The source of circulating ANGPTL-4 has not been elucidated. Based on pre-clinical and clinical studies, adipose tissue has been postulated as a relevant contributor to systemic ANGPTL-4 [1,2]. As ANGPTL-4 is released in three different forms, this is an important aspect to be considered while performing commercial assays. As we wanted to study the role of ANGPTL-4 in pediatric obesity, the kit we used recognized the full length of ANGPTL-4 (mainly secreted from adipose tissue) to detect if the amount of adipose tissue might influence its circulating levels in the pediatric population.

### Limitations of the Study

The main limitation of the present study is the sample size. In the cross-sectional study, the most limiting factor was the enrolment of normal weight participants. The clinical unit involved in the enrolment of the patients was specialized in pediatric obesity. However, the normal weight individuals were recruited by the gastroenterology unit where they were tested for food intolerance. In the longitudinal study, it was hard to find patients that could be followed up for two years. Furthermore, achieving weight loss in this period was difficult. Despite the low number of patients recruited in this study, the statistical analysis applied took into account the low sample size.

## 5. Conclusions

This study reveals that ANGPTL-4 circulating levels decrease in pediatric obesity, independent of the gender and pubertal stage, and are associated with the main markers of adiposity. In addition, the main biochemical parameters altered in obesity (FFA, cholesterol, vitamin D, etc.) are correlated with ANGPTL-4 levels. Further, in interventions achieving weight loss in children, the percent change of plasma ANGPTL-4 is positively associated with the percent change of FFA and negatively with the percent change of total cholesterol and HDL cholesterol. These results support the notion that ANGPTL-4 levels were associated with obesity and lipid profile in the pediatric population.

## Figures and Tables

**Table 1:** Anthropometric and biochemical characteristics of the cross-sectional study population.

	Lean (n = 73)	Obesity (n = 77)	*p*	Test
Age (year)	10.53 (3.45)	11.16 (3.07)	0.24	$
Sex (girl:boy)	38:35	40:37	0.99	
Sexual development (prepuberty:puberty)	34:39	38:39	0.73	
Birth weight (kg)	3.24 (0.63)	3.25 (0.55)	0.91	$
**Weight (kg)**	**38.70 (15.03**)	**62.64 (20.86)**	**<0.001**	#
**Height (cm)**	**140.85 (18.46)**	**148.39 (16.03)**	**0.01**	**$**
**BMI (kg/m^2^)**	**18.64 (3.63)**	**27.56 (4.59)**	**<0.001**	#
**Waist circumference (cm)**	**67.89 (12.47)**	**91.15 (13.27)**	**<0.001**	**$**
**Glucose (mg/dL)**	**83.86 (9.02)**	**79.12 (11.76)**	**0.007**	**$**
**Insulin (mUI/L)**	**6.72 (6.68)**	**13.83 (9.69)**	**<0.001**	#
**HOMA index**	**1.40 (1.29)**	**2.76 (2.12)**	**<0.001**	#
IGF-1 (ng/mL)	298.95 (184.69)	327.26 (189.30)	0.51	$
**Triglycerides (mg/dL)**	**55.14 (23.84)**	**65.24 (31.55)**	**0.03**	**$**
Free fatty acids (mg/dL)	12.37 (5.40)	11.37 (3.94)	0.41	#
Total cholesterol (mg/dL)	171.15 (36.25)	160.49 (31.07)	0.06	$
LDL-cholesterol (mg/dL)	102.26 (35.13)	97.24 (29.72)	0.37	$
**HDL-cholesterol (mg/dL)**	**59.57 (13.55)**	**46.60 (12.05)**	**<0.001**	**$**
**Leptin (ng)**	**9.65 (14.28)**	**18.30 (12.26)**	**<0.001**	**$**
TSH (mUI/L)	2.39 (1.18)	2.68 (1.15)	0.14	$
**fT4 (ng/dL)**	**1.08 (0.22)**	**1.16 (0.13)**	**0.03**	#
fT3 (pg/mL)	4.26 (0.46)	4.19 (0.40)	0.47	$
Estradiol (pg/mL)	31.12 (36.35)	26.13 (33.16)	0.41	$
Testosterone (ng/mL)	0.73 (1.43)	0.46 (0.67)	0.14	#
FSH (UI/L)	2.48 (2.16)	2.96 (2.55)	0.57	#
**Vitamin-D (ng/mL)**	**23.37 (12.38)**	**16.97 (6.59)**	**0.006**	#

Values are presented as mean (SD). Differences in sex and sexual development distribution were analyzed by *X^2^* analysis. Differences between groups were analyzed by t-test ($) or Wilcoxon test (#). Bold values mean significant statistical differences. BMI, body mass index; FSH, follicle-stimulating hormone; HOMA index, homeostasis model assessment of insulin resistance index; IGF-1, insulin-like growth factor 1; TSH, thyroid-stimulating hormone; fT3, free triiodothyronine; fT4, free thyroxine.

**Table 2:** Estimated coefficients (β) of the multivariate linear regression model of the cross-sectional study population, with standard error (SE), t statistic and *p* values.

	β (SE)	t Statistic	*p* Value
Intercept	59.65 (8.87)	6.72	<0.001
Obesity diagnostic	−14.86 (2.75)	−5.39	<0.001
Free fatty acids	0.75 (0.27)	2.77	0.007
LDL-cholesterol	0.15 (0.07)	2.15	0.03
Vitamin-D	0.04 (0.13)	0.28	0.77
Total-cholesterol	−0.15 (0.07)	−2.18	0.03

**Table 3:** Longitudinal effects on anthropometric and biochemical characteristics in participants from interventional study.

	Baseline	Follow-up	*p*
N	20		
**Age (year)**	**10.9 (2.6)**	**13.11 (2.6)**	**0.01**
Sex (girl:boy)	11:9		
Sexual development (prepuberty:puberty)	9:11	4:16	0.09
Weight (kg)	60.2 (18.2)	55.8 (12.3)	0.37
Height (cm)	147.8 (15.6)	156.9 (13.1)	0.05
**BMI (kg/m^2^)**	**26.8 (3.4)**	**22.3 (2.2)**	**<0.001**
**Fat mass (kg)**	**27.2 (10.0)**	**19.6 (6.3)**	**0.01**
Non-fat mass (kg)	29.5 (8.5)	32.9 (9.3)	0.27
**Percent Fat mass**	**45.3 (4.5)**	**32.7 (7.1)**	**<0.001**
**Waist circumference (cm)**	**89.4 (12.5)**	**81.7 (9.0)**	**0.03**
Glucose (mg/dL)	81.4 (9.0)	82.25 (7.9)	0.77
Insulin (mUI/L)	9.0 (5.2)	9.5 (6.7)	0.74
HOMA index	1.8 (1.0)	2.0 (1.4)	0.71
IGF-1 (ng/mL)	367.4 (197.2)	362.2 (179.3)	0.94
Triglycerides (mg/dL)	68.9 (55.8)	69.3 (32.0)	0.98
Free fatty acids (mg/dL)	13.5 (6.4)	14.9 (7.0)	0.52
Total cholesterol (mg/dL)	166.8 (25.4)	168.3 (30.4)	0.86
LDL-cholesterol (mg/dL)	102.6 (21.5)	101.6 (26.9)	0.90
HDL-cholesterol (mg/dL)	44.3 (8.7)	48.6 (12.5)	0.21
T-cholesterol/HDL	3.9 (1.2)	3.6 (1.0)	0.44
LDL/HDL	2.4 (0.9)	2.2 (0.9)	0.47
Leptin (ng/mL)	18.2 (20.9)	14.3 (17.4)	0.55
TSH (mUI/L)	2.7 (1.4)	2.5 (1.1)	0.63
fT4 (ng/dL)	1.1 (0.1)	1.2 (0.1)	0.11
fT3 (pg/mL)	4.1 (0.3)	4.1 (0.4)	0.86
Estradiol (pg/mL)	37.6 (56.3)	48.4 (79.4)	0.64
Testosterone (ng/mL)	0.6 (1.1)	0.6 (0.8)	0.93
FSH (UI/L)	3.3 (2.7)	3.1 (2.5)	0.80
Vitamin-D (ng/mL)	26.4 (16.5)	23.1 (14.7)	0.52
ANGPTL4 (ng/mL)	59.7 (4.0)	67.7 (4.1)	0.16

Values are presented as mean (SD). Differences in sex and sexual development distribution were analyzed by X^2^ test. Differences between groups were analyzed by paired T-test. Bold values mean significant statistical differences. BMI, body mass index; FSH, follicle-stimulating hormone; HOMA index, homeostasis model assessment of insulin resistance index; IGF-1, insulin-like growth factor 1; TSH, thyroid-stimulating hormone; fT3, free triiodothyronine; fT4, free thyroxine.

## Supplementary Materials

The following are available online at https://www.mdpi.com/2072-6643/11/6/1340/s1, Table S1: Anthropometric and biochemical characteristics of girls and boys of the cross-sectional study. Table S2: Anthropometric and biochemical characteristics of children and adolescents in the cross-sectional study. Table S3: Specificity study of ELISA kit. Table S4: Relationships of ANGPTL-4 with anthropometric and biochemical parameters measured in the cross-sectional study population. Table S5: Relationships of ANGPTL-4 with anthropometric and biochemical parameters measured in lean and obese subjects of the cross-sectional study. Table S6: Correlations between the percent change of ANGPTL-4 and the percent change of anthropometric and biochemical characteristics in participants from interventional study. Table S7: Estimated coefficients (β) of the multivariate linear regression model of the longitudinal study, with standard error (SE), t statistic and P values.
