# Supplementary material for: ANGPTL-4 is Associated with Obesity and Lipid Profile in Children and Adolescents
Source: Nutrients. 2019 Jun 14;11(6):1340. doi: 10.3390/nu11061340 (PMC6628529; doi:10.3390/nu11061340)
Supplement: Supplementary file 1 [file nutrients-11-01340-s001.pdf]

Table S1. Anthropometric and biochemical characteristics of girls and boys of cross-sectional study.

	Girls				Boys			
	**Lean (n= 38)**	**Obesity (n= 40)**	***P***	***Test***	**Lean (n= 35)**	**Obesity (n= 37)**	***P***	***Test***
Age (yr)	10.3 (3.5)	11.8 (3.2)	0.06	$	10.7 (3.4)	10.5 (2.8)	0.72	$
SD	17:21	17:23	0.84		17:18	21:16	0.49	
Birth weight (kg)	3.3 (0.4)	3.2 (0.5)	0.79	$	3.2 (0.8)	3.3 (0.5)	0.74	$
**Weight (kg)**	**41.3 (16.4)**	**63.7 (17.8)**	**<0.001**	**$**	**35.9 (13.0)**	**61.4 (24.0)**	**<0.001**	**#**
**Height (cm)**	**141.4 (17.7)**	**149.7 (14.1)**	**0.02**	**$**	140.2 (19.5)	146.8 (18.0)	0.15	$
**BMI (kg/m^2)**	**19.7 (4.0)**	**27.8 (4.4)**	**<0.001**	**$**	**17.5 (2.8)**	**27.2 (4.8)**	**<0.001**	**#**
**WC (cm)**	**69.2 (13.8)**	**91.8 (11.7)**	**<0.001**	**$**	**65.7 (10.7)**	**90.4 (15.0)**	**<0.001**	**#**
**Glucose (mg/dl)**	**83.9 (8.5)**	**77.2 (14.3)**	**0.01**	**$**	83.8 (9.7)	81.2 (7.6)	0.21	$
**Insulin (mUI/l)**	**8.2 (8.5)**	**15.0 (9.6)**	**0.003**	**$**	**5.1 (3.4)**	**12.5 (9.7)**	**0.001**	**#**
**HOMA-IR**	**1.70 (1.6)**	**2.9 (2.0)**	**0.01**	**$**	**1.1 (0.8)**	**2.6 (2.2)**	**0.003**	**#**
IGF-1 (ng/ml)	355.4 (186.1)	379.6 (209.3)	0.72	$	250.5 (175.5)	265.0 (141.7)	0.77	$
TG (mg/dl)	59.7 (20.3)	65.5 (26.1)	0.28	$	50.3 (26.5)	64.9 (37.0)	0.06	$
FFAs (mg/dl)	11.74 (5.3)	10.68 (4.1)	0.33	$	13.1 (5.4)	12.1 (3.6)	0.40	$
TC (mg/dl)	170.1 (32.8)	160.2 (23.6)	0.19	#	172.2 (40.1)	160.8 (38.0)	0.22	$
LDL-C (mg/dl)	102.0 (27.9)	96.5 (27.5)	0.41	$	102.5 (41.6)	98.1 (32.3)	0.63	$
**HDL-C (mg/dl)**	**56.7 (13.6)**	**46.5 (13.0)**	**0.002**	**$**	**62.3 (13.1)**	**46.7 (10.9)**	**<0.001**	**$**
**Leptin (ng/ml)**	**13.1 (17.7)**	**21.6 (12.7)**	**0.02**	**$**	**5.2 (5.8)**	**14.6 (10.7)**	**<0.001**	**#**
**TSH (mUI/l)**	2.5 (1.3)	2.5 (1.0)	0.96	$	**2.3 (0.9)**	**2.9 (1.3)**	**0.04**	**#**
**fT4 (ng/dl)**	1.1 (0.2)	1.2 (0.1)	0.40	#	**1.0 (0.2)**	**1.2 (0.1)**	**0.02**	**#**
fT3 (pg/ml)	4.2 (0.4)	4.1 (0.4)	0.72	$	4.4 (0.5)	4.3 (0.4)	0.50	$
Es (pg/ml)	45.4 (45.8)	36.9 (42.3)	0.43	$	17.3 (14.4)	14.1 (8.4)	0.27	$
T (ng/ml)	0.3 (0.2)	0.2 (0.2)	0.51	$	1.1 (1.9)	0.7 (0.9)	0.32	#
FSH (UI/l)	3.4 (2.4)	4.0 (2.6)	0.32	$	1.5 (1.3)	1.8 (1.8)	0.87	#
**VD (ng/ml)**	**25.0 (13.8)**	**16.2 (5.7)**	**0.008**	**#**	21.4 (10.5)	17.7 (7.4)	0.14	$

Values are presented as mean (SD). Differences in sexual development distribution were analyzed by X^2 analysis. Differences between groups were analyzed by t-test ($) or Wilcoxon test (#). Bold values indicate significant statistical differences. BMI: body mass index; Es: estradiol; FFAs: free fatty acids; FSH: follicle-stimulating hormone; HDL-C: HDL-cholesterol; HOMA-IR: homeostasis model assessment of insulin resistance; IGF-1: insulin-like growth factor 1; LDL-C: LDL-cholesterol; SD: sexual development (prepuberty:puberty); T: testosterone; TC: total cholesterol; TG: triglycerides; TSH: tyroid-stimulating hormone; fT3: free triiodothyronine; fT4: free thyroxine; VD: vitamin D; WC: waist circumference.

Table S2. Anthropometric and biochemical characteristics of children and adolescents subjects of cross-sectional study.

	**Children**				**Adolescents**			
	**Lean (n= 34)**	**Obesity (n= 38)**	***P***	***Test***	**Lean (n= 39)**	**Obesity (n= 39)**	***P***	***Test***
Age (yr)	**7.7 (2.3)**	**8.7 (2.0)**	**0.04**	**$**	13.0 (2.1)	13.5 (1.8)	0.27	$
Sex (girls:boys)	17:17	17:21	0.65		21:18	23:16	0.65	
Birth weight (kg)	3.1 (0.7)	3.3 (0.4)	0.41	$	3.3 (0.4)	3.2 (0.6)	0.46	$
**Weight (kg)**	**26.3 (9.1)**	**46.5 (11.7)**	**<0.001**	**$**	**48.4 (11.1)**	**77.6 (15.6)**	**<0.001**	**#**
**Height (cm)**	**125.8 (15.5)**	**135.2 (11.7)**	**0.007**	**$**	**152.7 (10.0)**	**160.5 (7.5)**	**<0.001**	$
**BMI (kg/m^2)**	**16.1 (1.9)**	**25.0 (3.3)**	**<0.001**	**$**	**20.6 (3.5)**	**29.9 (4.4)**	**<0.001**	**$**
**WC (cm)**	**58.8 (8.2)**	**82.4 (9.8)**	**<0.001**	**$**	**75.0 (10.5)**	**99.2 (10.7)**	**<0.001**	**$**
**Glucose (mg/dl)**	**82.4 (8.0)**	**76.9 (13.3)**	**0.04**	**$**	85.0 (9.7)	81.2 (9.8)	0.08	$
**Insulin (mUI/l)**	**4.1 (3.0)**	**9.7 (7.9)**	**0.001**	**#**	**9.0 (8.1)**	**18.0 (9.6)**	**<0.001**	**#**
**HOMA-IR**	**0.86 (0.7)**	**1.9 (1.8)**	**0.01**	**#**	**1.8 (1.5)**	**3.6 (2.1)**	**<0.001**	**#**
IGF-1 (ng/ml)	167.2 (133.2)	221.2 (143.3)	0.29	$	381.3 (165.5)	427.3 (173.4)	0.37	$
TG (mg/dl)	52.9 (26.1)	61.0 (30.0)	0.24	$	56.9 (22.0)	69.2 (32.8)	0.05	$
FFAs (mg/dl)	14.1 (6.0)	12.7 (3.9)	0.26	$	10.8 (4.3)	10.1 (3.5)	0.41	$
TC (mg/dl)	173.6 (44.9)	161.9 (35.7)	0.23	$	169.1 (27.7)	159.1 (26.3)	0.10	$
LDL-C (mg/dl)	106.0 (44.8)	100.4 (30.9)	0.55	$	99.1 (24.8)	94.3 (28.7)	0.45	$
**HDL-C (mg/dl)**	**62.4 (13.6)**	**45.5 (10.3)**	**<0.001**	**$**	**57.3 (13.2)**	**47.6 (13.5)**	**0.003**	**$**
**Leptin (ng/ml)**	**3.0 (3.5)**	**14.1 (9.0)**	**<0.001**	**#**	**13.8 (16.7)**	**22.3 (13.7)**	**0.02**	**$**
TSH (mUI/l)	2.5 (1.3)	2.6 (1.1)	0.54	$	2.3 (1.0)	2.7 (1.2)	0.14	$
fT4 (ng/dl)	1.1 (0.2)	1.2 (0.1)	0.07	#	1.1 (0.2)	1.1 (0.1)	0.20	#
fT3 (pg/ml)	4.3 (0.5)	4.2 (0.3)	0.32	$	4.1 (0.4)	4.2 (0.4)	0.94	$
**Es (pg/ml)**	**24.7 (24.1)**	**14.3 (9.7)**	**0.02**	**#**	35.9 (43.0)	38.0 (43.0)	0.83	$
T (ng/ml)	0.2 (0.1)	0.2 (0.4)	0.86	$	1.3 (1.9)	0.7 (0.8)	0.75	#
**FSH (UI/l)**	1.5 (1.5)	1.4 (1.7)	0.78	$	**3.2 (2.3)**	**4.5 (2.3)**	**0.02**	$
**VD (ng/ml)**	22.0 (12.7)	19.3 (6.6)	0.80	#	**24.1 (12.3)**	**14.5 (5.7)**	**<0.001**	#

Values are presented as mean (SD). Differences in sexual development distribution were analyzed by X^2 analysis. Differences between groups were analyzed by t-test ($) or Wilcoxon test (#). Bold values indicate significant statistical differences. BMI: body mass index; Es: estradiol; FFAs: free fatty acids; FSH: follicle-stimulating hormone; HDL-C: HDL-cholesterol; HOMA-IR: homeostasis model assessment of insulin resistance; IGF-1: insulin-like growth factor 1; LDL-C: LDL-cholesterol; T: testosterone; TC: total cholesterol; TG: triglycerides; TSH: tyroid-stimulating hormone; fT3: free triiodothyronine; fT4: free thyroxine; VD: vitamin D; WC: waist circumference.

Table S3. Specificity study of ELISA kit.

	Sample 1		Sample 2	
	Normal PL	Preadsorbed PL	Normal PL	Preadsorbed PL
**ANGPTL-4**	68.89 ng/ml	< 0.51 ng/ml	153.41 ng/ml	1.81 ng/ml

PL, plasma.

Table S4. Relationships of ANGPTL-4 with anthropometric and biochemical parameters measured in the cross-sectional study population.

	**ANGPTL4**	
	r	*P*
**Age**	-0.01	0.89
**Tanner state**	0.02	0.83
**Birth weight**	0.16	0.08
**Weight**	**-0.19**	**0.02**
**Height**	-0.07	0.37
**BMI**	**-0.27**	**0.001**
**Waist circumference**	**-0.25**	**0.002**
**Glucose**	0.02	0.77
**Insulin**	**-0.26**	**0.002**
**HOMA-IR**	**-0.27**	**0.002**
**IGF-1**	-0.07	0.49
**Triglycerides**	**-0.22**	**0.008**
**Free fatty acids**	**0.23**	**0.005**
**Total cholesterol**	0.02	0.85
**LDL-cholesterol**	-0.00	0.93
**HDL-cholesterol**	0.15	0.09
**Leptin**	**-0.19**	**0.03**
**TSH**	-0.03	0.71
**fT4**	-0.00	0.98
**fT3**	0.12	0.27
**Estradiol**	0.10	0.23
**Testosterone**	0.08	0.38
**FSH**	-0.03	0.75
**Vitamin D**	**0.24**	**0.009**

Statistical significance is from Pearson (normally distributed data) or Spearman (non-normally distributed data) correlation tests. Bold values indicate significant statistical differences. BMI: body mass index; FSH: follicle-stimulating hormone; HOMA-IR: homeostasis model assessment of insulin resistance; IGF-1: insulin-like growth factor 1; TSH: tyroid-stimulating hormone; fT3: free triiodothyronine; fT4: free thyroxine.

Table S5. Relationships of ANGPTL-4 with anthropometric and biochemical parameters measured in lean and obese subjects of the cross-sectional study.

	**ANGPTL4**			
	**Lean**		**Obesity**	
	r	*P*	r	*P*
**Age**	0.10	0.40	-0.11	0.32
**Tanner stage**	0.21	0.09	-0.07	0.57
**Birth weight**	0.21	0.11	0.10	0.45
**Weight**	0.14	0.26	-0.04	0.76
**Height**	0.11	0.37	-0.13	0.27
**BMI**	0.13	0.29	-0.05	0.65
**Waist circumference**	0.14	0.26	-0.00	0.96
**Glucose**	-0.08	0.50	-0.16	0.17
**Insulin**	0.13	0.30	-0.06	0.62
**HOMA-IR**	0.09	0.47	-0.09	0.44
**IGF-1**	-0.04	0.84	-0.07	0.56
**Triglycerides**	-0.06	0.61	**-0.27**	**0.02**
**Free fatty acids**	0.22	0.06	**0.34**	**0.002**
**Total cholesterol**	0.09	0.47	**-0.25**	**0.02**
**LDL-cholesterol**	0.18	0.15	-0.15	0.20
**HDL-cholesterol**	-0.02	0.88	-0.21	0.07
**Leptin**	0.22	0.10	-0.13	0.28
**TSH**	0.09	0.44	-0.01	0.92
**fT4**	0.23	0.08	-0.10	0.41
**fT3**	-0.13	0.55	0.20	0.12
**Estradiol**	0.14	0.29	0.02	0.84
**Testosterone**	0.11	0.49	-0.00	0.95
**FSH**	0.18	0.16	-0.13	0.28
**Vitamin D**	-0.04	0.82	0.13	0.27

Statistical significance is from Pearson (normally distributed data) or Spearman (non-normally distributed data) correlation tests. Bold values mean significant statistical differences. BMI: body mass index; FSH: follicle-stimulating hormone; HOMA-IR: homeostasis model assessment of insulin resistance; IGF-1: insulin-like growth factor 1; TSH: thyroid-stimulating hormone; fT3: free triiodothyronine; fT4: free thyroxine.

Table S6. Correlations between the percent change of ANGPTL-4 and the percent change of anthropometric and biochemical characteristics in participants from interventional study.

	**% change of ANGPTL4**	
	r	*P*
Percent change of age (%)	-0.14	0.56
Percent change of weight (%)	0.01	0.97
Percent change of height (%)	-0.02	0.93
Percent change of BMI (%)	0.06	0.79
Percent change of fat mass (%)	-0.13	0.63
Percent change of non fat mass (%)	-0.00	0.98
Percent change of % fat mass (%)	0.45	0.16
Percent change of waist circumference (%)	0.18	0.45
Percent change of glucose (%)	-0.38	0.10
Percent change of insulin (%)	-0.08	0.78
Percent change of HOMA index (%)	-0.41	0.88
Percent change of IGF-1 (%)	0.00	0.99
Percent change of triglycerides (%)	-0.19	0.41
**Percent change of free fatty acids (%)**	**0.67**	**0.001**
**Percent change of total cholesterol (%)**	**-0.45**	**0.04**
Percent change of LDL-cholesterol (%)	-0.25	0.28
**Percent change of HDL-cholesterol (%)**	**-0.51**	**0.02**
% change of total cholesterol/HDL	0.22	0.35
% change of LDL/HDL	0.26	0.27
Percent change of leptin (%)	0.25	0.37
Percent change of TSH (%)	0.15	0.54
Percent change of fT4 (%)	0.43	0.11
Percent change of fT3 (%)	0.42	0.12
Percent change of estradiol (%)	-0.36	0.15
Percent change of testosterone (%)	0.37	0.21
Percent change of FSH (%)	-0.00	0.99
Percent change of vitamin D (%)	-0.22	0.35

Statistical significance is from Pearson correlation test. Bold values indicate significant statistical differences. BMI: body mass index; FSH: follicle-stimulating hormone; HOMA: homeostasis model assessment of insulin resistance; IGF-1: insulin-like growth factor 1; TSH: tyroid-stimulating hormone; fT3: free triiodothyronine; fT4: free thyroxine.

Table S7. Estimated coefficients (β) of the multivariate linear regression model of longitudinal study, with standard error (SE), t statistic and P values.

	**β (SE)**	**t statistic**	**P value**
**Intercept**	22.20 (7.93)	2.80	0.01
**Percent change of free fatty acids**	0.16 (0.04)	4.35	0.001
**Percent change of HDL-C**	-0.35 (0.21)	-1.64	0.12
**Percent change of total cholesterol**	-0.43 (0.40)	-1.08	0.30
**Percent change of triglycerides**	-0.05 (0.06)	-0.89	0.39

HDL-C: HDL-cholesterol.
